# A perspective for biomedical data integration: Design of databases for flow cytometry

**DOI:** 10.1186/1471-2105-9-99

**Published:** 2008-02-14

**Authors:** John Drakos, Marina Karakantza, Nicholas C Zoumbos, John Lakoumentas, George C Nikiforidis, George C Sakellaropoulos

**Affiliations:** 1 Department of Medical Physics, School of Medicine, University of Patras, GR-26504 Rion, Greece; 2 Haematology Division, Department of Internal Medicine, School of Medicine, University of Patras, GR-265 04 Rion, Greece

## Abstract

**Background:**

The integration of biomedical information is essential for tackling medical problems. We describe a data model in the domain of flow cytometry (FC) allowing for massive management, analysis and integration with other laboratory and clinical information. The paper is concerned with the proper translation of the Flow Cytometry Standard (FCS) into a relational database schema, in a way that facilitates end users at either doing research on FC or studying specific cases of patients undergone FC analysis

**Results:**

The proposed database schema provides integration of data originating from diverse acquisition settings, organized in a way that allows syntactically simple queries that provide results significantly faster than the conventional implementations of the FCS standard. The proposed schema can potentially achieve up to 8 orders of magnitude reduction in query complexity and up to 2 orders of magnitude reduction in response time for data originating from flow cytometers that record 256 colours. This is mainly achieved by managing to maintain an almost constant number of data-mining procedures regardless of the size and complexity of the stored information.

**Conclusion:**

It is evident that using single-file data storage standards for the design of databases without any structural transformations significantly limits the flexibility of databases. Analysis of the requirements of a specific domain for integration and massive data processing can provide the necessary schema modifications that will unlock the additional functionality of a relational database.

## Background

The integration of biomedical information has become an essential task for health care professionals. Current progress in the domain of Information Technology allows for huge data storages and powerful computational possibilities to be affordable; thus, they have been quite common. Researchers are gradually becoming aware of the importance of keeping together diverse data pertaining to a specific medical entity and successful attempts to create and maintain such databases are becoming known to the scientific community [1].

Data models specified by standards are often included in databases, without taking into account inherent limitations posed by the procedure of acquiring original data. It is therefore quite often that inference is affected by errors that propagate throughout the entire process, from data acquisition through processing and analysis. While data models are adequate for their initial usage, they are inflexible to the requirements posed by new analysis procedures that become available as data are massively aggregated from diverse origins. Often, they include data reduction steps, narrowing the scope of future analyses. Having the data in their raw form, however, would offer the opportunity of reanalyzing the data as new, unpredicted at the time of acquisition, hypotheses are put under test.

Integration is therefore much more than a plain collection of digital biomedical data [2]. Homogenization of data description and storage, followed by normalization across the various experimental conditions would be a prerequisite for facilitating procedures of knowledge extraction [3].

### The Flow Cytometry Standard (FCS)

**Figure 1:**
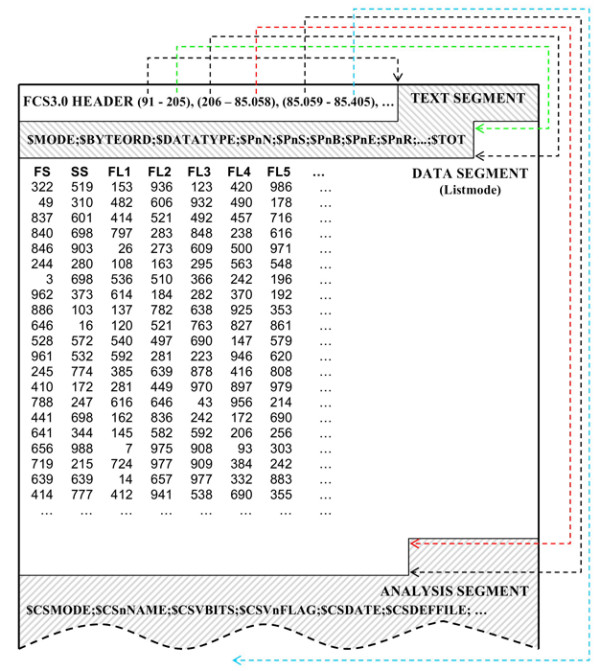
**The structure of FCS files**. The HEADER is a fixed size (58 bytes), ASCII encoded segment, which always occurs at the beginning of an FCS file. The first six bytes of each header are occupied by FCS' version (e.g. "FCS3.0"). The rest of the header describes the location of the other segments in the data files ('text seg. begin', 'text seg. end', 'data seg. begin', 'data seg. end', 'analysis seg begin' and 'analysis seg. end'). The HEADER may be extended beyond 58 bytes, if custom segments exist in a FCS file. The TEXT segment is divided in 'primary' and 'supplemental' sub-segments, containing a series of ASCII encoded keyword-value pairs that describe various aspects of the data and the conditions under which the experiment took place. The DATA segment contains the raw data, consisting of a number of light-intensity measurements (varying from 6 to 34, in most cases) for each passing cell, depending on the particular cytometer's technology. The raw data are stored in one of four allowed formats ('integer', 'floating point', 'double precision floating point' or 'ASCII'). The mode, format, bit length and all other necessary information needed to extract the raw data from an FCS file are stored in the TEXT segment.

The FCS is a data storage protocol [4], proposed by International Society for Analytical Cytometry (ISAC), aiming to provide a transparent way of storing data produced by flow cytometers. Files produced according to FCS have three obligatory segments ('HEADER', 'TEXT' and 'DATA') which may be followed by an optional 'ANALYSIS' segment and other, manufacturer defined, custom segments (Figure 1).

The TEXT segment is divided in two sub-segments, 'primary' and 'supplemental', and contains a series of ASCII encoded keyword-value pairs that describe various aspects of the data. The 'primary' sub-segment contains a fixed, obligatory set of keywords while 'supplemental' sub-segment is optional and contains a variable number of keywords according to the level of details each hardware manufacture implements on each flow cytometer. Additional to describing the data produced during an experiment (flow cytometry measurement), the conditions under which the experiment took place can be recorded.

The DATA segment contains the raw data in one of three modes (list, correlated, uncorrelated). The majority of flow cytometers store data in list-mode integer format. List-mode data storage means that events (cells) are stored one after the other in a list. The data consist of a number of light-intensity measurements for each passing cell, depending on the particular cytometer's technology.

### Vertical & Horizontal database schemata

Usually, data file storage standards pay much attention to the fidelity aspect of data storage: to provide the conditions so that data are exported from the acquisition devices in a uniform manner, to form reproducible images of the examinations performed. They do not take providence of integration with similar data originating either from different acquisition hardware or from different installations. However, it is often the case that efficient data management requires non-intuitive forms of data structures; when data integration is sought, it is the Information Technology (IT) principles that pose the optimality criteria.

### Horizontal information level

We define the term 'Horizontal Information Level' (HIL) as the data of a single record of a database table or the record formed by joining database tables in a single step that produces unique records. In other words, the participating tables should be related with primary – foreign keys. The use of word "Horizontal" was chosen to visualize that a HIL always attains the form of a row.

Every HIL may be assigned to a real life concept. In our test case of flow cytometry (FC) a record from the table storing the FCS header segment is a HIL (header HIL) and it represents information about the experiment and the patient. Another HIL (cell HIL) is a record from the table storing the FCS data segment and it represents the measurements concerning a single cell. Joining the header and cell HILs creates a new one, representing antigen values measured for a specific cell of a specific patient in a specific examination (measurement HIL). The group of measurement HILs from all the records having the same experiment ID represents a specific experiment (experiment HIL); the group of experiment HILs having the same patient ID represents patient's history and so on.

### Vertical Database Schema

We define the term 'Vertical Database Schema' as the database design in which the retrieval of all data originating from the same HIL is not enough to render them useful. For data to become useful, data-mining actions must be applied in one or more fields of the HIL. Since these actions are specific to each field that needs mining and common for all its data (visually: a column), we characterize such actions vertical.

**Figure 2:**
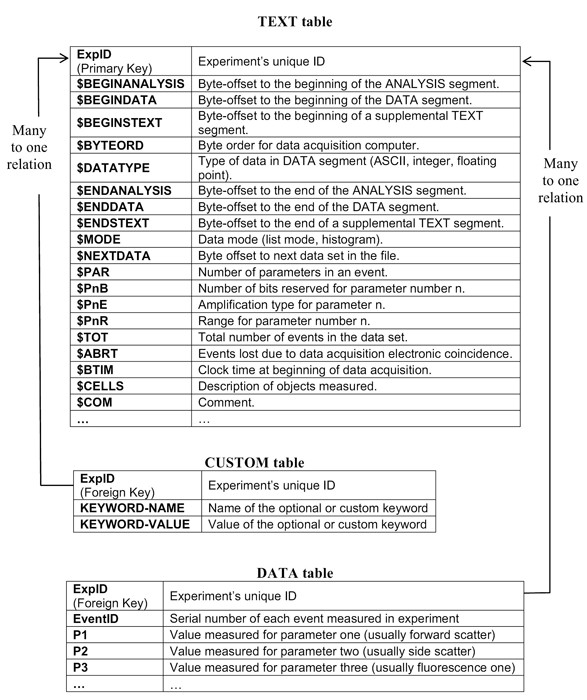
**A first attempt for an efficient conventional schema**. The FCS standard allows for optional and custom keywords that differ from one file to another. To accommodate custom keywords that differ from vendor to vendor, we could add a 'CUSTOM' table containing the keyword name – keyword value pairs of all custom parameters met in the FCS files. This table would be related to the 'TEXT' and 'DATA' tables through the 'ExpID' primary – foreign key relation. You can notice that measurements are stored in the generic fields P1, P2 etc. Each time data is retrieved from one of these fields, extra actions must take place to identify which specific antigen is measured and to align all antigen measurements under the same field.

**Figure 3:**
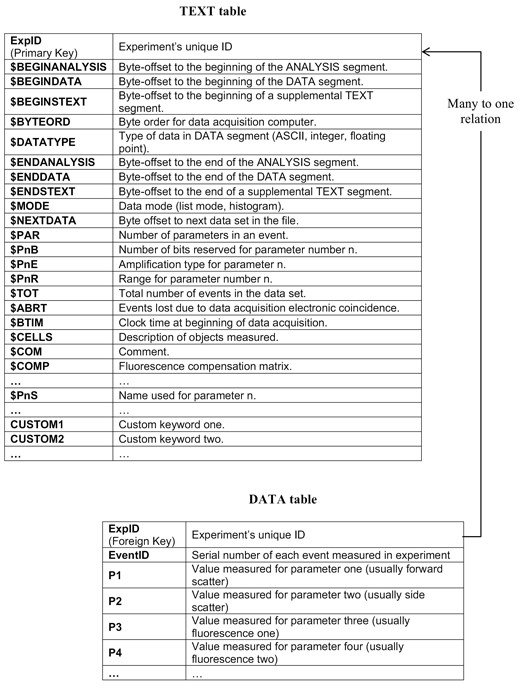
**A second attempt for an efficient conventional schema**. The conventional schema that was adopted for reference and comparisons. Following a pre-analysis of all FCS files with regard to the custom parameters used, we could obtain the entire set of keyword names for the FCS files under consideration, and include them in the 'TEXT' table structure as we did with the optional FCS keywords. The procedure of TEXT segment analysis must be repeated before any data import can take place.

A database storing FCS data with a schema that is an exact replica of the standard's structure (Figures 2 &3) is a clear example of a Vertical Database Schema. The 'measurement HIL', for example, cannot be used before some data-mining actions take place. To make cell measurements meaningful (fields P1, P2, etc), a check must be made for the parameter number under which each value is measured. Then the description of that parameter (fields P1S, P2S, etc) must be used to identify the antigen that this parameter represents in the specific experiment. Knowing that P1 has the value of 512 has no particular meaning before identifying that P1 is measuring side scatter or log of side scatter or CD20.

Furthermore, to retrieve data from different 'experiment HILs', two new data-mining actions must be applied. One is to identify all the different name forms that various sources may have used for the same antigen (e.g. CD20, CD-20, CD/20 etc) and unify them into a single name (e.g. CD20). The other is to check whether different fields, from several experiments, contain measurements of the same antigen (e.g. CD20 measurements are contained in field P3 in experiment one and in P6 in experiment nine). In such cases more data-mining actions must take place to align all data originating from the same antigen under a unique field. It's meaningless to retrieve files co-expressing P2/P3 when what P2 and P3 stand for is not clear.

At this point it is important to clarify that holdbacks of a Vertical Database Schema following the FCS file structure originate from the database design, not FCS itself. Using a standard created for single-file data storage as a blueprint for building an integration host for data originating from different sources consists a problematic approach. Within the boundaries of a single file none of the aforementioned problems occur.

### Horizontal Database Schema

**Figure 4:**
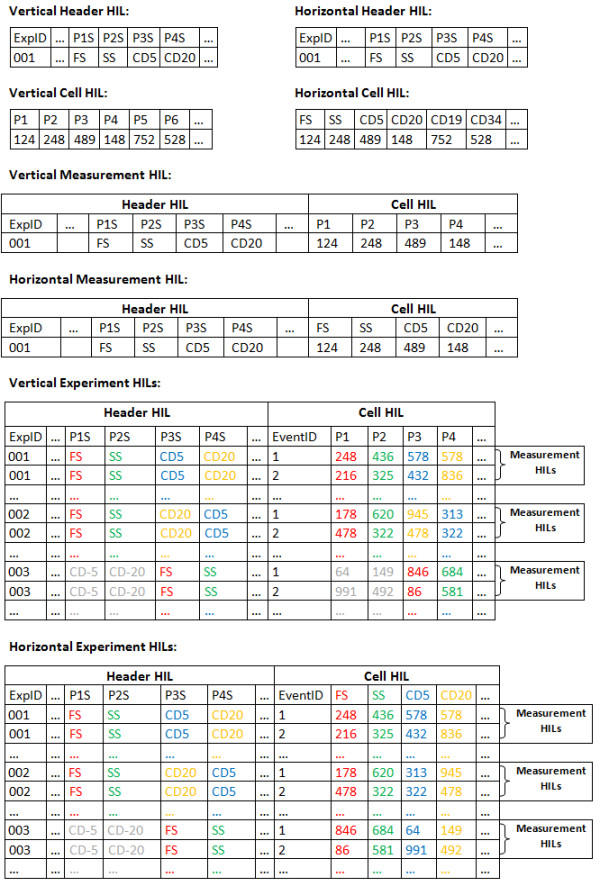
**Horizontal vs Vertical**. Header HILs: similar to both schemata. Cell HILs: While in horizontal HILs the fields have clear meanings (FS, SS, CD5 etc), vertical HILs are useless because field labels cannot be resolved. Measurement HILs: In the horizontal case, cell fields maintain their clear meanings and HIL gains value from the additional fields of the Header segment. In the vertical case, the additional information of the Header segment can be used to resolve the cell field labels. Even though both HILs contain the same amount of information, it remains hidden in the vertical case. Experiment HILs: Horizontal HILs manage to remain simple (all fields have clear meanings and data is perfectly aligned among different records) despite the fact that the amount of information has significantly grown. For vertical HILs to become useful, the user must resolve field labels, unify the different naming forms of the same antigen and finally align all the fragmented values of the same antigen under a single field.

We define the term "Horizontal Database Schema" as the database design in which the retrieval of all data originating from the same HIL is enough to render them useful. No further data mining is needed (Figure 4) for the retrieved information to become useful. Horizontal Database Schemata are vital when intra- or inter-domain integration is the goal of an information system.

In the following we evaluate the serious inflexibilities of the Vertical Database Schema when data integration on the level of the specific antigen measured, patient or population of patients is our goal. The database schema that we propose alleviates these disadvantages, offering the possibility of storing the data in a horizontal manner that allows for data integration and analysis.

### The (problematic) conventional database schema

The conventional way to store FCS data in a relational database is to reproduce the FCS protocol structure with database tables and then fill these tables without any kind of structural transformation.

A first approach to implementing this schema would be to create a database consisting of two tables. The 'TEXT' table's fields would be the keywords appearing in the TEXT segment of the FCS file. Each record in this table would correspond to one experiment and a primary key field 'ExpID' would be necessary to identify individual experiments. The DATA' table would contain the actual measurements; its fields would be the parameters measured, using their generic names (P1, P2 etc). Modern cytometers record measurements of up to 256 parameters (in some extreme cases) per event. Each record would correspond to a specific cell measured within a specific experiment. The field 'ExpID' would also be present here, as a foreign key to the corresponding field of 'TEXT' table. Within each experiment, every cell should be identified by a serial number, so one additional field 'EventID' would be required. The combination of fields 'ExpID' and 'EventID' will act as the primary key of the 'DATA' table.'

The problematic character of this approach comes directly from the fact that the FCS standard allows for optional and custom keywords that differ from one file to another. Optional fields are specified in the FCS standard, while custom keywords differ from vendor to vendor. While we can include all optional FCS fields in the 'TEXT' table structure, knowing that most of them would remain blank, we cannot a priori know the variety of custom fields' nomenclature.

We can think of two possible attempts to alleviate this drawback. The first (Figure 2) would be to have an additional 'CUSTOM' table containing the keyword name – keyword value pairs of all custom parameters met in the FCS files. This table would be related to the 'TEXT' and 'DATA' tables through the 'ExpID' primary – foreign key relation. A second attempt (Figure 3) would call for a pre-analysis of all FCS files with regard to the custom parameters used. Had we at our disposal the entire set of keyword names for the FCS files under consideration, we could include them in the 'TEXT' table structure as we did with the optional FCS keywords. The procedure of TEXT segment analysis must be repeated before any data import can take place.

This latter approach (Figure 3) was adopted as the conventional schema for reference and comparisons. While such a schema may be satisfactory for a simple recording of FCS data, it is not suitable for queries. What a medical doctor or a researcher expects from databases for FC data is the ability to perform queries that would answer real-life questions. Queries of the type "which patients express a specific antigen above a predefined level" or "what is the distribution of a specific antigen expression in a specific group of patients", regardless of the hardware and/or environment within the experiment took place.

Unfortunately, the conventional database schema presented above is unable to produce results from such queries since, by design, it maintains a vertical logic.

### Aims of the paper

This paper aims at presenting:

• The design of a relational database model that follows the Horizontal Database Schema and is optimal with respect to its ability to support complex, large scale and quick response queries

• The development of an application that imports data from FCS files and exports them to the relational database

• The evaluation of the data model and the application through real-life inquiries. The evaluation is based on measurements of the complexity and length of the queries compatible with the conventional and the proposed schema and of the time needed for the queries to be processed.

## Results

The design of the new relational database model was based on the requirements of end-users, namely clinicians and researchers in the field of FC. To this end, an extensive collaboration between physicians, computer scientists and medical physicists took place. Two of the co-authors (MK & NCZ), physicians in charge of the application of flow-cytometry techniques in the Patras University Hospital, surveyed the Haematologists working with flow cytometry within the University Hospital. The survey did not follow a systematic approach but attempted to cover all current research and routine requirements of the staff. The database should provide the end-user with the possibility of performing syntactically simple and intuitive queries pertinent to scientific questions emerging in clinical routine as well as in research. The data should be kept in a way as transparent as possible, allowing for easy integration with clinical data as well as with data originating from other laboratory modalities. Finally, the design should be optimized for the management of massive data, with respect to the information kept per examination, the magnitude of existing FCS data file repositories and the need for fast results on complex queries.

### The database schema

**Figure 5:**
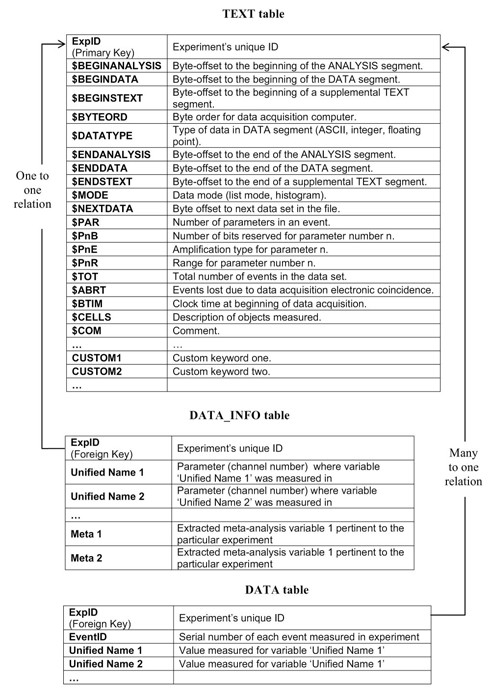
**The proposed database schema**. The proposed schema consists of three tables. Under this schema, data acquired on every HIL is useful, without the need for extra data-mining actions. For example, all measurements of antigen CD20 are stored in a unique field (e.g. 'Unified Name 2').

The database schema we propose (Figure 5) is able to fulfil these requirements because of its clear horizontal structure. During our extensive testing, no query or HIL of any level contained data requiring further processing to become meaningful.

It consists of the following tables:

1. A TEXT table, containing all keyword-value pairs of the FCS primary and supplemental sub-segment. An additional numeric field 'ExpID' is the primary key to this table.

2. A DATA table, containing the actual measured values. 'ExpID' is also used as foreign key here, not unique this time. A new field, 'EventID', is added to characterize each individual event (cell) recorded by the cytometers. The combination of 'ExpID' and 'EventID' is used as the primary key of the 'DATA' table to discriminate among different cells and experiments.

The 'DATA' table contains one field for each quantity measured across the entire database. For example, all measurements regarding antigen CD34 are stored in a single field. 'Null' entries (blanks) reflect the lack of measurement of the corresponding quantity in the particular event.

3. A DATA_INFO table, containing the channel number that a particular parameter was measured in each experiment. It has identical structure to the 'DATA' table, and since it refers to experiments rather than cells, a field characterizing each cell (EventID) is not needed. 'ExpID', is again a foreign key to the corresponding field of TEXT table but this time it is unique. 'DATA_INFO' table acts as an accelerator to identify which experiments measure specific parameters (antigens) and under which wavelength. If, for example, CD20 was measured in a specific experiment in channel 8 (P8) then the record for this experiment would contain the value 8 under the field CD20. This way, even though all measurements referring to antigen CD20 are stored under the same field of 'DATA' table, the user is able to identify different flavours of the antigen (FITC, PE, Cy5, etc). In the future, DATA_INFO might also contain experiments' metadata, e.g. the average expression of an antigen.

The 'ExpID' field is indexed on all tables to accelerate combined searches. The proposed schema can be implemented in any relational database supporting basic table relations (primary – foreign keys). Since imported data is counted in millions of rows it is not suggested to store FC data on databases without indexing capabilities.

### The application

To simplify and accelerate data import for the proposed schema, a specially designed application was developed. The application is able to massively import flow cytometry data that follow the FCS standard irrespective of the hardware's specifications and manufacturer.

The application's main concern is the homogenization of the variables' names, since the FCS standard permits the user to give any name to the measured parameters. The homogenization is performed through a graphical interface or by user-defined sets of rules (in the form of regular expressions). Additionally, in occasions where the FC operator does not specify the name of the corresponding variable, and the parameter takes default names (e.g. "FL1"), the application can extract the variable name from alternative TEXT segment fields (e.g. the examination title, if present) using the same regular expressions (e.g. search for CD5 in a title like "CD20/CD5 test").

The main algorithm was developed having in mind the widest possible scope of application and flexibility. Instead of using "hard-wired" statements and thus being bound to currently known implementations by FC makers, our algorithm parses and interprets the header of any data file following the FCS 3.0, 2.0 or 1.0 standard. Hence, it dynamically allocates the necessary fields for the export procedure, according to the specifications read from the file's TEXT segment. As a result, it is able to read any FCS file not being limited by FC hardware specification regarding quantization level, word length, byte order, amplification, number of channels etc.

### Quantitative results

For the evaluation of both the proposed database schema and the application, 30.201 FC datafiles acquired during the period 2000–2006 at the Patras University Hospital were imported to two databases, following the conventional (Figure 3) and the proposed schema (Figure 5), respectively. Most of the preliminary query tests revealed that the conventional database did not respond within reasonable time or did not respond at all due to hardware limitations. We therefore restricted the datafile range to patients belonging to a specific disease (B-chronic lymphocytic leukaemia). 3.741 FCS datafiles corresponding to 534 examinations and involving measurements of 43 antigens were massively parsed. 109 variants in the naming of the various antigens were recorded. In total, the TEXT table, storing FCS datafile header information, contained 3.741 records, while the DATA table, storing raw cell data, contained 71.623.106 records. The numbers of records are the same in both compared database schemata, since we applied structural and not data transformations.

The evaluation consisted of the following steps: First, having in mind the common tasks for clinicians within the context of chronic lymphocytic leukaemia (CLL), we analyzed the retrieval process of experiments co-expressing specific antigens. For this purpose, several queries were prepared, simultaneously measuring 2, 3 or 4 fluorescent wavelengths (colours), to cover the entire spectrum of possible requirements (Table 1). Second, we checked the horizontal logic of the proposed database tables and their relations by verifying the advantages of all horizontal HILs mentioned in Figure 4.

**Table 1:** The parameters inquired

Query	Parameters inquired	Fluorescent wavelengths
1	FS, SS	2, 3 or 4
2	FS, SS, CD5	2, 3 or 4
3	FS, SS, CD5, CD20	2, 3 or 4
4	FS, SS, CD5, CD20, CD19, CD34	4

Each inquiry was transformed into a suitable query for the two database schemata, the proposed and the conventional, taking into account the variability in hardware design.

### Complexity of queries

In our proposed database schema, transforming our research inquiries into suitable queries is straightforward. The queries are small and resemble the natural language. Additionally, they do not depend on the number of colours used in a specific experiment or are supported by the FC. For example, the syntax for query 2 (see Table 1) would be:

**SELECT **ExpID, FS, SS, CD5 **FROM **DATA **WHERE **ExpID **IN**

(**SELECT **ExpID **FROM **Data_Info **WHERE **CD5 **IS NOT NULL**)

**ORDER BY **ExpID

The conventional schema, on the other hand, gives rise to extremely lengthy and complex queries, not allowing the user to easily follow the steps involved. The exact syntax of query 2 on the conventional database, for an experiment concerning two colours would occupy two pages of this article. Queries on the conventional database heavily depend on the specific hardware; they grow in length as the number of colours measured increases, as shown in Table 2.

**Table 2:** Query length (conventional schema vs. proposed)

Query	Conventional schema			Proposed schema
	Two colours	Three colours	Four colours	

1	1,142	1,390	1,638	96
2	5,138	7,762	10,946	123
3	12,526	19,774	28,702	150
4	N/A	N/A	89,558	204

The length (in bytes) of the queries created for the conventional schema depends on the number of colours supported by the hardware, and is significantly higher when compared to the proposed schema. The 'Query' column identifies the queries of Table 1.

**Figure 6:**
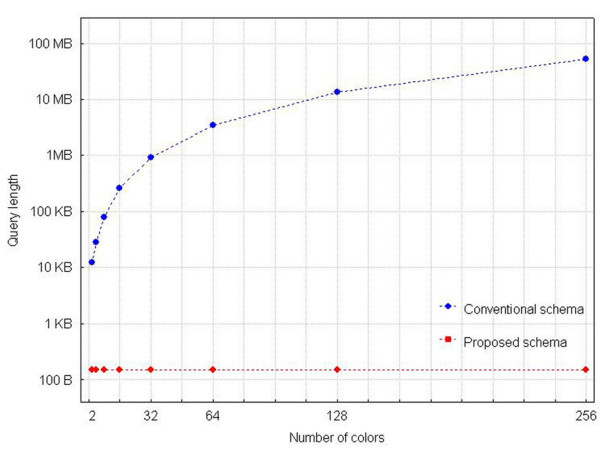
**Dependence of Query 3 complexity on the specific FC hardware**. The complexity of Query 3 (see Table 1) as a function of colours supported by the specific FC hardware. The size of the queries for the proposed schema remains constant and several orders of magnitude smaller than those for the conventional schema.

The dependence of the complexity of Query 3 on the specific hardware is shown in Figure 6. While the query length within the proposed database schema is constant throughout the range of possible colours supported (150 bytes), queries for the conventional schema grow quickly out of manageable size (e.g. 938 KB for 32 colours) due to the data-mining operations needed.

To further investigate the magnitude of the difference in query length for equivalent queries applied to the two database schemata, we estimated the corresponding lengths for a number of queried quantities, up to a maximum number of 256. For these estimations, we took under account the 'worst-case' scenario, i.e. that we seek the values of k quantities among k measured quantities (query 4, mentioned earlier, serves as an example for k = 4).

**Figure 7:**
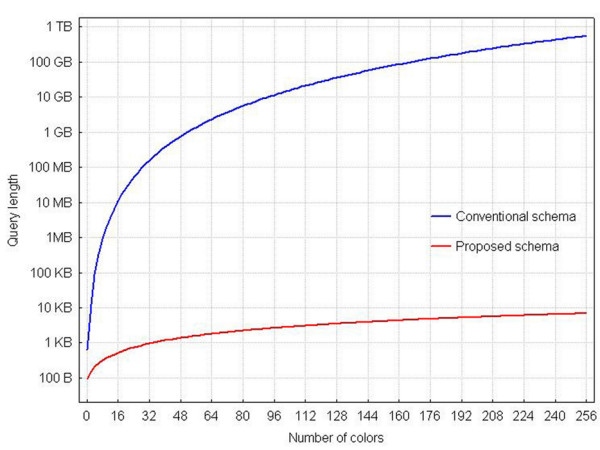
**Comparison of complexity between the two schemata**. Query length as a function of the number of colours taken into account for retrieval. For a given number of colours (k), we enquire the measured values of all events (cells) for k variables. Indicative values (conventional, proposed): (11.2 Mb, 528 b) for k = 16, (158 Mb, 960 b) for k = 32, (2.33 Gb, 1.8 Kb) for k = 64. The value k = 0 corresponds to retrieval of forward scatter (FS) and side scatter (SS) values only.

Figure 7 graphically exhibits these results. Evidently, the query length for the conventional schema quickly acquires magnitudes beyond the handling abilities of users. Queries on the proposed database schema, on the other hand, never exceed 7,000 characters, for the entire range of cytometers known to date.

### Database schema efficiency

Three users simultaneously submitted the same queries to the database. Using the logging capabilities of the database server, we measured both the CPU time allocated for each query and the time elapsed between query submission and completion (response time). Since the queries concern a varying number of database records, we normalized our measurements and selected the time per million records as a robust indicator of database schema efficiency.

**Figure 8:**
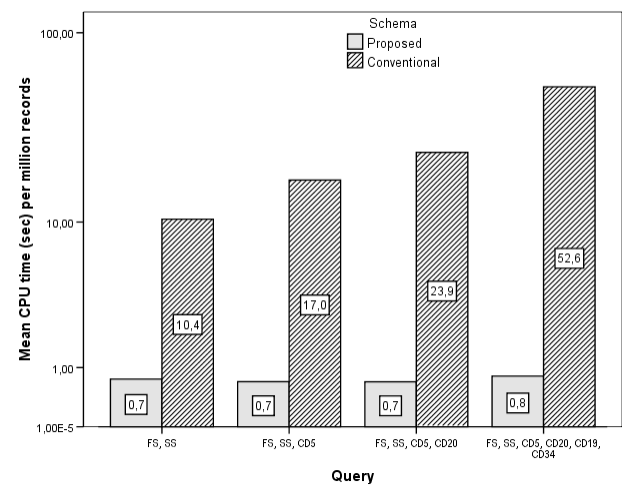
**CPU times**. CPU times for various queries under the proposed and conventional database schema.

**Figure 9:**
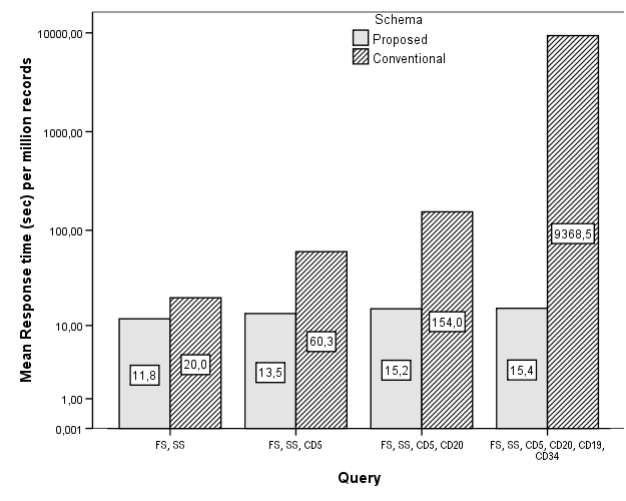
**Response times**. Response times for various queries under the proposed and conventional database schema.

The average CPU and response times are shown in Figures 8 and 9 (logarithmic scale). It is evident that the response times are significantly lower for the proposed schema, a direct consequence of the smaller query length.

It is also evident that in real world databases with hundreds of millions of records, the times needed under the conventional schema for relatively simple queries (like query 4), become forbiddingly long.

### Potential for integration with other biomedical data

**Figure 10:**
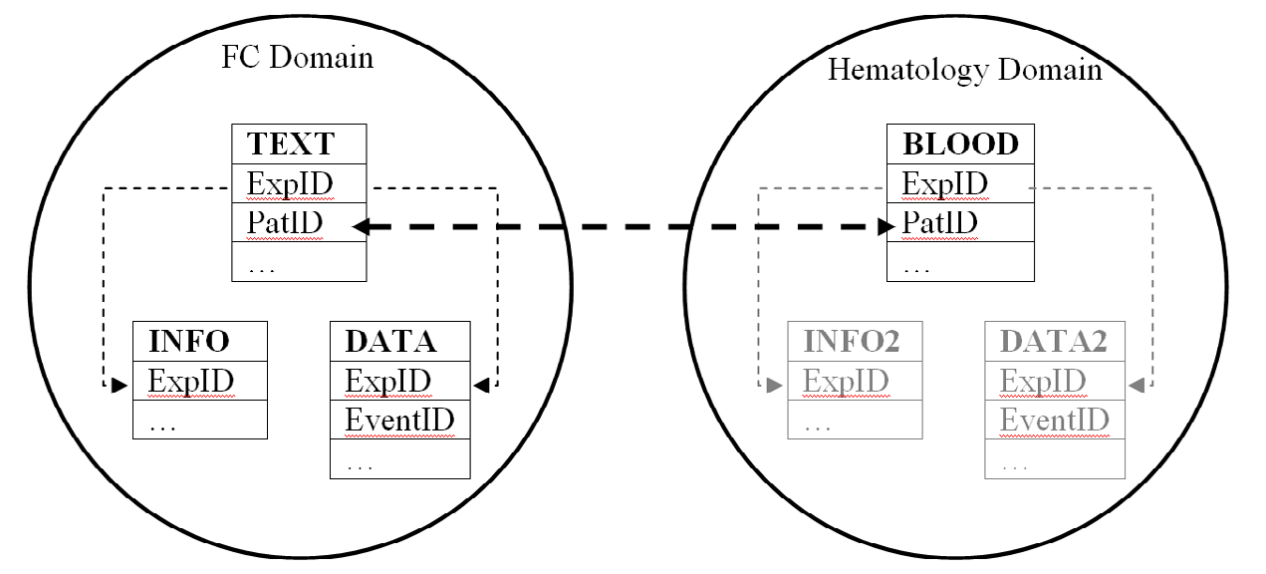
**Integration with other biomedical data**. The layout of a scenario for integration with data from the Haematology domain. Given that the BLOOD table is designed under the principles of our proposed schema, the two domains are easily integrated through an appropriate unique field (Patient ID)

The horizontal logic behind the proposed database schema can be applied to other types of medical data. In the following, we present a scenario for integration with data from the Haematology domain (Figure 10).

An extra table (BLOOD) is added to the database to make storage of haematological data possible. The BLOOD table must have a clear horizontal structure. If haematological data are not gathered in a cumulative form (e.g. number of lymphocytes and lymph nodes), but in a more detailed fashion (e.g. characteristics of each individual lymph node) then one more table is needed to store the detailed information and keep the horizontal structure of the database schema (following the TEXT-DATA tables of the FC example). An accelerator table may be added if required by the number of detailed haematological records. The accelerator table will help data processing by indicating if a variable is measured or not, in a specific experiment, before starting the data retrieval process.

Patient ID, a unique number assigned to each patient (on a hospital, region or country level) would provide a safe passage between data originating from different domains. Using the patient's name instead, would introduce many false positives and overall low accuracy. In addition, it offers better control of sensitive personal information. FCS datafiles used in our test database contain, as a custom TEXT variable, a patient ID.

Having unique identifiers for all stored variables and keeping haematological information in a single unified manner, like in the FC example, allows maintaining a horizontal schema among inter-domain medical data. A possible query to retrieve data from the integrated database, "find FC patient records measuring CD5, CD20, CD19, CD 34, having high lymphocyte count and high number of lymph nodes" would be formulated as:

**SELECT **TEXT-ExpID **FROM **TEXT **JOIN **BLOOD **ON **TEST-PatID = BLOOD-PatID **WHERE **(CD5 **IS NOT NULL**) **AND **(CD20 **IS NOT NULL**) **AND **(CD19 **IS NOT NULL**) **AND **(CD34 **IS NOT NULL**) **AND **(LCYTES > 10.000) **AND **(LNODES > 6)

Even though the above query is bigger than the previously presented examples concerning purely FC data, it is still maintains a simple form, easy to read and fast to execute. Had the integrated database been built following a vertical schema, the effort to create and run joint queries would have been prohibiting and would require computational power and memory beyond the available capabilities at most installations.

## Discussion

FC, in addition to standard molecular examinations, can be used in multiplexed testing environments [5], since it can contribute a top-down resolution of the molecular biocomplexity [6] and insights on how difference in gene expression translates into cell differentiation and function. From the Informatics point of view, integrating FC data can thus be considered as a step in the growing synergy between Bioinformatics and Medical Informatics [7]. Under this meaning, integration of biomedical data means much more than obtaining the data in digital form or even reducing diverse data to a common form (e.g. as tables in Microsoft Excel files). Easily accessible integrated data are therefore regarded to be a central and key issue [8,9] and there are now attempts to develop databases that contain both clinical and genetic data [10-12].

FCS being a file storage standard, poses no particular specifications regarding integration. Our horizontal database model concerns the massive collection of datafiles from multiple locations. The two by no means compete; we believe that our model provides additional value to the FCS by inheriting all the variables and properties of a FCS datafile (cytometer's characteristics, patient data, institution data etc) and keeping the data in a schema that is efficient in terms of drawing conclusions from a large collection of such files.

Current implementations of FC databases [13-17] mainly provide facilities for data visualization and extraction of parametric values on a per examination basis. In their recent article, Cavenaugh et al. [18] describe a relational database for immunological clinical trials, a part of which concerns flow cytometry. They store eight FCS keywords and seven meta-data variables extracted from preliminary analyses performed by an external application (FCS Express V.2.0). Our proposed database schema can be viewed as an improvement to their work. It is capable of storing the entire spectrum of FCS defined variables, either obligatory, optional or custom. In addition, the lowest level of cytometric information is stored, allowing flexibility for any possible analysis or integration with data originating from other medical domains. While this kind of information is recognized by Cavenaugh et al. as unnecessary within the context of their article, we believe that it becomes a big asset when designing open-architecture databases.

Popular commercial software, like CellQuest (by Becton Dickinson) and WinList (by Verity Software House), could benefit by storing the acquired data in a database following the proposed schema and not only in separate files. Instead of being limited to operations similar to "open file Z0001234.lmd", they would have the capability for operations like "open files of 'Patient XX' measuring 'CD5/CD20"'.

The database has been tested by physicians of the Haematology Department as a tool for their research and their feedback has already been incorporated in the model we present. Formulating the queries has not been a problem due to the human-near language syntax used and the small length that the queries attain under our schema. However, we are currently working on the development of a (web) interface that will provide the users a graphical environment for even easier query formulation. Then the end-users will incorporate it in their routine clinical work and additional feedback will be gathered.

Uniformity of data identifiers and the additional effort required to achieve it during data entry, can be considered as a limitation of our proposed data model. However, this should be compared against the much larger cumulative cost of dealing with naming inhomogeneities every time the data are used. A minor disadvantage of our model is the increase of storage space needed, since most records do not use the entire spectrum of the available variables and null values are stored in the corresponding table columns. We believe increased disk space is only a minor disadvantage, mainly for three reasons: (1) mass storage capabilities are available to most modern computer systems and are much cheaper than CPU power, (2) increasing the storage space by a factor of two (as measured in our database) is a well justified cost to pay for a decrease of response times by orders of magnitude, and (3) using many tables and a "vertical" architecture, in an effort to reduce null-value entries, renders the database unusable because of increased complexity.

## Conclusion

Quite often, efficient data management requires non-intuitive forms of data structures; when data integration is sought, optimality criteria differ from those emerging from a mere representation of the data acquisition procedure into a database. Integration requires "horizontal" data structures, where the entire information of a given HIL is ready to use after the retrieval process. The database schema that we propose endorses principles that allow for massive data integration. Regardless of the number of heterogeneous sources, originating from a single or multiple domains, it maintains manageable levels of complexity and provides the possibility for analyses that can be orders of magnitude faster than conventional approaches.

## Methods

Quantitative assessments of the benefits gained by our proposed schema, as well as measurements of the robustness of performance, were performed in a controlled computer network environment consisting of three personal computers of varying computer power and a database server. The databases presented were hosted by Microsoft SQL Server 2005™. The application was developed in Visual Basic for Applications (VBA) and implemented as an add-on of Microsoft Excel.

## Availability and requirements

All queries can be found in our web site .

## Authors' contributions

JD and GCS conceived of the study, and participated in its design. JD carried out the data analysis, performed all necessary measurements and assisted in writing of the manuscript. MK and NCZ surveyed the end-user requirements and gathered their feedback. MK, NCZ and JL participated in the acquisition of the list mode data and the interpretation of the results. GCN participated in the design of the study and assisted to draft the manuscript. GCS coordinated the study and wrote the manuscript. All authors read and approved the final manuscript.
